# Surgical Practice in Resource‐Limited Settings: Perspectives of Medical Students and Early Career Doctors: A Narrative Review

**DOI:** 10.1002/hsr2.70352

**Published:** 2025-01-14

**Authors:** BillSmith Anyinkeng Achanga, Christian Wabene Bisimwa, Victor Oluwafemi Femi‐Lawal, Nnoko Sona Akwo, Tohson Falake Toh

**Affiliations:** ^1^ Department of Biomedical Science University of Buea Buea Cameroon; ^2^ Department of Cardiothoracic Surgery Alexandria University Alexandria Egypt; ^3^ College of Medicine University of Ibadan Ibadan Nigeria; ^4^ Department of Occupational and Environmental Health University of Buea Buea Cameroon; ^5^ Department of Clinical Medicine University of Buea Buea Cameroon

**Keywords:** early career doctors, limitations, low‐ and middle‐income countries, medical students, surgical practice

## Abstract

**Introduction:**

Surgical practices in low‐resource countries often fail to meet established standards. Both doctors and medical students have limited exposure to surgical cases, which hinders training and the development of surgical specialization. This study highlights the current state of surgical practice from a trainee's perspective, explores existing gaps in training and capacity building, and recommends practical solutions.

**Methods:**

We conducted a literature search on PubMed, Google Scholar, and other scientific databases using search terms such as “surgical practice,” “doctors' perspectives in surgical practice,” “surgery in low‐ and middle‐income countries,” and “solutions to surgical inadequacy.” We included studies published from 2015 to 2024, with exceptions for a few highly relevant studies published prior to 2015.

**Results:**

We outline the limitations identified in the literature concerning surgical training and healthcare in low‐ and middle‐income countries. Many centers lack adequate infrastructure, human resources, and training. These challenges negatively affect the skills and quality of surgical care. However, some centers demonstrate that surgical practice is feasible through collaboration with institutions established in higher‐income contexts.

**Conclusion:**

Telesurgery, task shifting and sharing, high‐impact, low‐cost surgeries, and collaborations with more developed health systems could effectively bridge the gap in surgical availability in LMICs.

## 1 Introduction

About one‐third of the global disease burden is surgical, highlighting its unique relevance in optimum patient care and global disease burden. These diseases cause 18 million deaths per year, most of which occur in resource‐limited settings [1, 2]. The Lancet Commission on Global Surgery (LCGS) estimates that 5 billion people lack access to safe, affordable surgical care, with low‐resource countries paying some of the highest costs [3]. Surgical interventions are often considered complex procedures to be undertaken only by highly trained specialists, but the cadres are rare in many low‐resource settings. The cases of South–East Asia and Africa, with only 12% of the world's surgical specialists (surgeons, anesthetists, and obstetricians), while harboring a third of the world's population, are important to consider. The density of specialist surgeons in these countries is only 0.7/100,000; a minimum density of 20/100,000 is considered necessary to tackle the burden of surgical disease [4].

Healthcare resources can be grouped into three broad categories: infrastructure, equipment, and human resources. In the context of surgical practice, a resource‐limited setting is one with poor infrastructure, inadequate materials, and insufficient qualified personnel to render good surgical care [5]. Studies have shown that up to 50% of surgical interventions in low‐ and middle‐income countries (LMICs) are performed in primary health centers or district hospitals [6]. In many of these societies, healthcare is virtually under governmental control. While the government is almost entirely responsible for infrastructure and equipment, its ability to provide human resources is very limited.

Universal health coverage is present in some LIMCs, but it is not fully implemented, as even the USA utilizes only 17% of its Gross National Income on health. Likewise, the three agendas that form the priorities of universal health coverage have yet to be fulfilled by them [1]. Most patients are still required to pay for their treatment on arrival at the hospital, and also cover supplies, medications, and surgical fees out‐of‐pocket.

Another challenge for surgical care services in resource‐limited settings is that the policies by which they are governed often do not align with the realities of low‐resource care provision. These challenges in resources, infrastructure, and training lead to significantly poorer patient outcomes, including mortalities and morbidities. Mughal et al., for example, report that adults undergoing emergency abdominal surgery in LMICs are three times more likely to die postoperatively than similar patients in high‐income countries [7]. Studies also show that 50% of deaths within 30 days of surgery are from low‐resource settings, despite much fewer surgeries occurring in these countries [8, 9]. These suboptimal outcomes are due to significant disparities in access to quality surgical care in these countries, occasioned by infrastructure, training, and personnel barriers.

To tackle the deficits in these settings, early career doctors and non‐specialists are often trained to perform certain crucial procedures traditionally in the domain of specialists. Task shifting and task sharing were established as an interim mechanism to plug the specialist gap but have since evolved to become the mainstay of surgical care delivery in many countries with limited resources [10, 11]. Surgeons in these countries acknowledge the utility of these solutions in tackling personnel shortages and optimizing patient care. The absence of essential resources to improve surgical practice and the quality of training received by medical students and early career doctors have led to significant gaps in the quality of care provided to patients, reducing life expectancy and making safe surgery a challenge. Given the unique position of surgical care in these settings, early career doctors and medical students are integral to improving the surgical workforce in resource‐limited settings.

In this study, we highlight the current state of surgical practice in resource‐limited settings such as LMICs and how it affects early career doctors' training and capacity building. By doing so, this study will contribute to the growing evidence base and provide relevant information to help policymakers and stakeholders make effective decisions for improving surgical practice and training outcomes in resource‐limited settings, ultimately improving patient outcomes.

## 2 Methods

To find relevant studies, we conducted a comprehensive search of databases such as PubMed and Google Scholar. Our search terms were related to “surgery,” “surgical practice,” “resource‐limited settings,” and “LMICs.” We optimized our search using Boolean operators, MESH terms, and field tags. Citations were then screened to determine eligibility for inclusion in the study.

### 2.1 Inclusion Criteria

We included studies that met the following criteria:

- Language and publication: articles written in English and published in peer‐reviewed journals.
- Time frame: studies published between 2015 and 2024, with exceptions for select pre‐2015 articles that addressed perennial challenges in surgical practice within resource‐limited settings.
- Focus: articles reporting on surgical practice in resource‐limited settings, specifically those addressing medical students' and early career doctors' challenges and perspectives.

### 2.2 Exclusion Criteria

We excluded:

- Non‐peer‐reviewed articles.
- Letters to the editor, commentaries, and opinion pieces.
- Studies unrelated to surgical practice in resource‐limited settings.

Each eligible study underwent a rigorous quality assessment to ensure relevance and reliability. This included evaluating the study design, data sources, and validity of findings. Studies that met these standards were included in the narrative synthesis.

## 3 Understanding the Perspective of Students and Early Career Doctors in Resource‐Limited Setting

### 3.1 Challenges Faced by Students and Early Career Doctors in Resource‐Limited Settings

In resource‐limited settings, early career doctors face a number of challenges with regard to surgical practice and training. These include:

#### 3.1.1 Lack of Access to Modern Surgical Equipment and Technology

In a recent review by Chu et al., they noted that most colon and rectal resections are done by open techniques, often because of the unavailability of equipment for laparoscopic or robotic procedures [12]. The lack of advanced surgical equipment is reported as a major barrier to surgical training [13, 14, 15]. Only the larger hospitals and academic institutions are able to support training in advanced techniques such as endoscopic surgery [16]. In countries like Brazil, trainees often have to wait till much later in their careers to acquire training in laparoscopic surgery [16, 17]. The lack of equipment is due to a myriad of factors, such as poor financing and insufficient transport systems [18]. Even when equipment is made available, it is often challenging for programs to sustain the training required. In Nigeria, a 5‐day laparoscopic training established by stakeholders from Europe was unsustainable due to financial constraints [19]. Delays in transport, for example, can jeopardize surgical practice.

#### 3.1.2 Limited Exposure to Diverse Surgical Cases

Surgical trainees in low‐income settings typically handle fewer cases than those in higher‐income countries [6]. In a recent study, South African surgeons estimated that 24 laparoscopic cholecystectomies needed to be performed by surgical trainees, but only 19.2 had been completed by the end of the training [14]. Time constraints, lack of supervision, and limited volume of cases have been reported as contributory factors [14, 15, 20, 21].

Limited exposure to cases may also be due to limited capacity to handle complex surgical cases. In Ethiopia, surgeons are often discouraged from performing laparoscopic surgery due to a paucity of qualified residents [20]. Chu et al. mention that laparoscopic and robotic procedures are less common in low‐income areas, potentially affecting the frequency and diversity of surgical cases in these facilities [22]. A Pakistan study revealed that the median distance traveled by patients to access surgical care for brain tumors was ~104 km [23]. Besides affecting the quality of care that patients receive, this significantly impacts access to surgical care. With minimal access to care, many challenging cases will likely not present at surgical facilities, affecting the richness of training.

Additionally, only a few centers are able to carry out certain procedures. In India, only two centers in big cities contribute to more than 50% of the 420 epilepsy surgeries carried out in the country [23]. While outcomes in these centers are sometimes comparable to those in high‐income settings [24, 25], most trainees will likely not have sufficient access to these cases to build their capacities. Cost is also a predisposing factor—insurance platforms are relatively underdeveloped in low‐resource settings; those in higher socioeconomic classes are more likely to obtain care [26].

#### 3.1.3 Educational Resource Constraints

Only a few centers in low‐resource settings have the capacity to train surgical residents to global standards. Watila et al. note that many established centers lack adequate infrastructure, personnel, and training [27]. Therefore, there is often a lack of trainers in remote LMIC regions [28]. However, Uganda shows that training is achievable through collaboration with established centers in higher‐income settings [29]. This is shown in centers such as the Charles Nicolle Hospital in Tunisia, Aga Khan University Hospital in Pakistan, and Shiraz University of Medical Sciences in Iran [30, 31, 32]. However, this may present problems with sustainability. In Bolivia and Nicaragua, time constraints limited training courses to a maximum of 5 days, potentially leading to mixed outcomes [33].

There is also a lack of variation in the quality and depth of training [15]. Training guidelines are often derived from HICs, and thus may be unsuitable in low‐resource settings [34]. These factors significantly impact trainees' competencies and skills.

#### 3.1.4 Limited Mentorship and Supervision of Students in Underserved Communities (USCs)

Many skilled medical trainees and specialists boycott rural areas for financial and comfort reasons. This further widens the equity gap to the benefit of urban areas [35]. In addition, USCs are very difficult to access due to long distances and poorly maintained transportation networks [36, 37]. These lead to challenges with providing surgical training in these localities.

## 4 Challenges in Surgical Practice Within a Resource‐Limited Setting

Excellent surgical care is an essential component of a functional health system [38]. Unfortunately, surgical care remains inaccessible to 5 billion people globally, particularly in LMICs, making the sustainable development goal of safe and timely access to surgery by 2030 difficult to achieve [39]. Providing access to safe, affordable, and timely surgical care is still a challenge in sub‐Saharan Africa, as only 15% of the need is currently met [40]. In addition, postoperative mortality is disproportionately high in LMICs. The African Surgical Outcomes Study (ASOS) in 2019 revealed that postoperative morality in this setting is twice as high as the global average [41]. In Cameroon, as of 2021, the rural population represented 41.85%, seeking healthcare primarily at first‐level healthcare facilities [39, 42]. These challenges may be attributable to several factors [34], which we will discuss below.

### 4.1 Lack of Infrastructure and Resources

Underserved communities (USCs) lack adequate infrastructure for surgical care, ranging from operating theaters to diagnostic equipment and trained personnel [35]. Inadequacies in the national budget quota for health expenditures, and poor governance are some of the few factors compromising these services [7]. Lack of basic needs like running water supply, electric power supply, and theater equipment renders the delivery of surgical care very challenging in centers where theaters do exist [43]. Furthermore, communities affected by crises have exposed several healthcare centers to destruction or occupation by military barracks. These infrastructural gaps make access to surgical care challenging [44].

### 4.2 High Patient Volume and Acuity

There is a shortage of skilled surgeons in several low‐resource communities. The few available surgeons face overwhelming workloads that negatively impact the efficiency and efficacy of the care that they provide. Many LMICs have a density of surgical specialists severely below the recommended minimum level (20/100,000 people) [41]. In addition, consistent brain drain toward developing countries contributes to these drops in the numbers of trained personnel, further contributing to increases in relative patient volume.

### 4.3 Limited Mentorship and Supervision of Students

There is limited mentorship and supervision of students practicing in USCs. For financial and comfort reasons, many skilled employees boycott rural areas. This further widens the equity gap to benefit urban areas [45]. This lack of available mentorship in these regions limits the ability of trained residents and surgeons to provide surgical care when needed [41, 46].

### 4.4 Poor Health Financing Structures and Lack of Health Insurance

The expensive nature of surgical care limits access to health care in rural areas [47, 48]. Therefore, out‐of‐pocket expenditures are the most common way to access complex surgical procedures [47]. Health insurance coverage is grossly insufficient in several resource‐limited countries, causing several patients to make catastrophic out‐of‐pocket payments in order to obtain surgical care [47]. These high costs drive many patients toward traditional healers, negatively impacting access to safe surgical care in large hospitals and academic healthcare establishments [49].

## 5 Discussion (Way Forward)

Inadequate surgical practice is still a significant public health burden across LMICs due to resource limitations, insufficient numbers of personnel, and poor infrastructure. Thus, immediate pathways to reduce these gaps are needed. We recommend the strategies below:

### 5.1 Incorporation of Telemedicine Into Surgical Care

Telemedicine with telesurgery is another great tool in enhancing surgical practice in resource‐limited settings. The first telesurgery campaign was carried out by the United Nations Special Forces in Somalia in 2001 and gave hope to the people in places with shortages in surgical resources, infrastructure, and personnel [50]. Between 2003 and 2005, about 21 assisted robotic telesurgery procedures were performed, including procedures inguinal hernia repairs and right hemicolectomies [51]. This could be a good ground to improve access to safe surgical care in Africa, improving cost savings, increasing follow‐up rates, and decreasing morbidity and mortality. However, poor internet connection and electricity shortages would pose a barrier to achieving this progress. To solve these challenges, governments and health institutions must invest significantly in ICT and telemedicine infrastructure, providing opportunities for telemedicine in pre‐ and postoperative visits, provider–provider consultations, and intraoperative mentoring. Studies of this nature have been reported in China, South Africa, and Turkey, and their models may be replicated in other LMICs [52].

### 5.2 Task Shifting and Task Sharing

Task shifting and task sharing will be effective ways to efficiently close the gaps relating to low surgical availabilities in LMICs. Thus, specialist surgeons may train early career healthcare professionals into becoming efficient surgeons who could reach out to the suburbs. Surgeons may also integrate these early career doctors into their surgical teams, frequently exposing them to surgical techniques for further expertise [53].

### 5.3 High‐Impact, Low‐Cost Strategies

The use of high‐impact, low‐cost strategies is highly recommended, implementing surgical capacities at the district hospital level, the first point of contact reducing excessive burden on the reference hospital. Their participation in enhancing surgical capacity in LMICs is pivotal, in association with other global health programs [54]. Incorporating telemedicine into these strategies also proves vital. In a recent study, Owolabi et al. revealed that telephone calls and two‐way texting were useful in patient management and follow‐up. Video calling was also useful in preoperative care and case management [52].

### 5.4 Surgical Mentorship Opportunities for Medical Students and Early Career Doctors

Medical student's involvement is crucial in reducing the lack of access to surgical care in LMICs, as they are the future surgeons and health providers. Early exposure by training in surgical care, surgical research, and mentorship could reduce the prominent brain drain in LMICs.

LMICs are also highly encouraged to partner with countries with more developed healthcare systems to improve their training, mentorship, and surgical outcomes. This will help build stronger mentor–mentee relationships, promoting the acquisition of surgical skills and more confident surgeons ready to face these challenges [55]. This approach helps integrate holistic patient care and accommodates the community's unique needs.

## 6 Conclusion

Surgical practice in resource‐limited countries is still below standard. However, the perspective of early career students and medical students is very vital, as they are the future of the surgical workforce. Thus, poor perceptions by medical students and early career doctors, inadequate exposure to surgical cases, and inadequate training can potentially affect the surgical workforce in the future. It is therefore important for governments, institutions, and international stakeholders to collaborate for innovation, research, and creating improved opportunities to improve surgical care and capacity in LMICs. Adopting these interventions can improve the quality and accessibility of surgical care in these contexts, enhance the quality of training, and ultimately lead to improved patient outcomes.

## Author Contributions

**BillSmith Anyinkeng Achanga:** writing–original draft, writing–review and editing, project administration, conceptualization. **Christian Wabene Bisimwa:** supervision, writing–review and editing. **Victor Oluwafemi Femi‐Lawal:** writing–original draft, project administration, writing–review and editing. **Nnoko Sona Akwo:** writing–original draft, writing–review and editing. **Tohson Falake Toh:** writing–original draft.

## Conflicts of Interest

The authors declare no conflicts of interest.

## Transparency Statement

The lead author Achanga Bill‐Smith Anyinkeng affirms that this manuscript is an honest, accurate, and transparent account of the study being reported; that no important aspects of the study have been omitted; and that any discrepancies from the study as planned (and, if relevant, registered) have been explained.

## Data Availability

The authors have nothing to report.
